# Higher Intraocular Pressure Levels Associated With Lower Hysteresis In Type 2 Diabetes

**DOI:** 10.2174/1874364101812010029

**Published:** 2018-03-28

**Authors:** Sinan Bekmez, Tolga Kocaturk

**Affiliations:** 1 Department of Ophthalmology, Dr. Behcet Uz Children’s Training and Research Hospital, Izmir, Turkey; 2 Department of Ophthalmology, Faculty of Medicine, Adnan Menderes University, Aydin, Turkey

**Keywords:** Ocular response analyzer, Corneal biomechanic properties, Type 2 diabetes mellitus, Corneal hysteresis, Intraocular pressure, Lower hysteresis

## Abstract

**Aim:**

To investigate the differences of corneal biomechanic characteristics using Ocular Response Analyzer (ORA, Reichert; USA) on type 2 diabetics and healthy subjects.

**Methods:**

One hundred eyes of 100 subjects (between the ages of 17-91) who applied to Adnan Menderes University’s Ophthalmology Clinic between January-March 2015 were included in this study, 50 diabetics (Group 1) and 50 healthy controls (Group 2). The eyes included in the study were randomly chosen. Corneal Hysteresis (CH), Corneal Resistance Factor (CRF), Goldmann correlated Intraocular Pressure (IOPg) and corneal compensated Intraocular Pressure (IOPcc) of patients were measured by ORA. Detailed ophthalmological examinations were done for every subject. Kolmogorov-Smirnov test was used to analyze the distribution of quantitative variables and t test was used for the data that were normally distributed. Any p value <0.05 was considered as statistically significant.

**Results:**

The mean ages were 63.3±9.0 and 61.7±11.6 in Group 1 and 2, respectively (p=0.459). 25 (50.0%) were female, 25 (50.0%) were male in Group 1 and 26 (52.0%) were female, 24 (48.0%) were male in Group 2 (p=1.000). Mean IOPcc values were 17.8±3.6 (12.1-29.0) and 16.0±3.1 (10.9-23.8) mmHg (p=0.006); mean IOPg values were 16.9±3.5 (10.9-25.9) and 15.4±2.9 (9.0-24.7) mmHg (p=0.032); mean CH values were 9.9±1.5 (6.1-13.3) and 10.5±1.7 (6.5-15.7) (p=0.080) and mean CRF values were 10.4±1.6 (7.5-14.0) and 10.5±1.7 (6.6-15.4) (p=0.730) in Groups 1 and 2, respectively.

**Conclusions:**

There was no any statistical difference between the groups in terms of CH and CRF. However, mean CH and CRF values were found less in diabetic group. Corneal biomechanical differences seen in diabetic patients may be associated with a statistically significantly higher IOP measurements.

## 1 INTRODUCTION

Ocular Response Analyzer (ORA, Reichert; USA), which is a non-contact tonometer, is the first device capable of dynamic evaluation of the in vivo biomechanical properties of the cornea [1]. The device sprays the air jet to form deformation in the cornea. ORA air pressure creates two cornea current response measurements (P1, P2) depending on the impact: The force required to flatten the cornea with rising pressure, and the force required to flatten the cornea again with decreasing pressure. The difference between the two pressures (P1-P2) is termed “Corneal Hysteresis” (CH). The average of the two applanation pressure is described as compatible with Goldmann IOP (IOPg). The device taking into account the CH determines a second IOP (IOPcc), compensated by the biomechanical properties of the cornea. The other important parameter of the device is a Corneal Resistance Factor (CRF) [2]. Clinical trials with ORA have shown that the device is not affected or slightly affected by CCT values [3].

Diabetes Mellitus (DM) is a systemic disease which affects the eye in very different ways: diabetic retinopathy, neovascular glaucoma, cataract, ptosis, oculomotorius nerve palsy [4]. Dry eye symptoms can typically be observed in diabetics, like burning and foreign body sensation, decreased in visual quality [4]. Besides corneal complications in diabetic patients, CCT was investigated. In some publications, there is no difference in CCT between diabetic patients and normal subjects [5, 6]. Many publications reported that CCT increased in diabetic patients [7-10].

The aim of this study was to investigate the differences in corneal biomechanic characteristics between the patients with and without type 2 DM.

## 2 METHODS

One hundred eyes of 100 subjects (between the ages of 17-91) who applied to university outpatient clinic between January-March 2015 were included in this study, 50 diabetics (Group 1) and 50 controls (Group 2). The eyes included in the study were randomly chosen. CH, CRF, IOPg and IOPcc of patients were measured by ORA.

The patient exclusion criteria of the study include: patients who have any corneal pathology, uveitis, and posterior segment pathology, lens pathology that prevent the fundus examination, dry eye or conjunctivitis is detected, ocular trauma and previous ocular surgery history, patients who underwent ocular intravitreal injection, patients who take any topical treatment, patients who get treatment medications for systemic disease except DM and patients who noncooperate to measure with ORA.

The study protocol had the approval of the university’s ethics committee and complied with the guidelines set forth in the Declaration of Helsinki. Detailed ophthalmological examinations were done for every subject.

### 2.1 Statistical Analysis

Kolmogorov-Smirnov test was used to analyze the distribution of quantitative variables and t- test was used for the data that were normally distributed. Any p value <0.05 was considered as statistically significant. If the data did not fit a normal distribution, Mann-Whitney-U test was used for comparisons between the groups. Descriptive statistics of normally distributed data were shown as mean ± standard deviation. Descriptive statistics were shown as the median (25-75 percentiles) for the data that did not fit a normal distribution. Chi-square test was used for qualitative data analysis and descriptive statistics were shown as frequency (percent). All results were analyzed statistically using the SPSS (Statistical Package for the Social Sciences; SPSS Inc., Chicago, IL, USA) version 16 software package for Windows.

## 3 RESULTS

The mean ages were 63.3±9.0 and 61.7±11.6 in Group 1 and 2, respectively (p=0.459). 25 (50.0%) were female, 25 (50.0%) were male in Group 1 and 26 (52.0%) were female, 24 (48.0%) were male in Group 2 (p=1.000) (Table **1**). Mean IOPcc values were 17.8±3.6 (12.1-29.0) and 16.0±3.1 (10.9-23.8) mmHg (p=0.006); mean IOPg values were 16.9±3.5 (10.9-25.9) and 15.4±2.9 (9.0-24.7) mmHg (p=0.032); mean CH values were 9.9±1.5 (6.1-13.3) and 10.5±1.7 (6.5-15.7) (p=0.080) and mean CRF values were 10.4±1.6 (7.5-14.0) and 10.5±1.7 (6.6-15.4) (p=0.730) in Groups 1 and 2, respectively (Table **2**). There was no statistical difference between the groups in terms of CH and CRF. However, mean CH and CRF values were found less no in the diabetic group. There was a statistically significant difference between the diabetic and nondiabetic groups in terms of IOP parameters. Mean IOPcc and IOPg values were found high in the diabetic group.

## 4 DISCUSSION

In our study, we investigated the differences of ORA measurements between healthy subjects and type 2 diabetic patients. Mean IOPcc and IOPg values were found to be statistically significantly high in the diabetic group, associated with lower CH and CRF levels.

The study of Scheler *et al.* investigated the relationship between HbA1c levels and ORA measurements in diabetic patients [11]. They revealed a higher rate of CH and CRF levels in poorly controlled diabetic patients. There wasn't any difference between the well-controlled diabetic patients and healthy control groups in biomechanical measurements in their study.

In Cankaya *et al.*’s study, they compared the biomechanic properties of the cornea between patients with and without type 2 diabetes [12]. IOPg, CRF and CCT values were statistically significantly higher in diabetic patients than healthy subjects. We revealed a higher rate of IOPcc and IOPg values in diabetic patients.

Corneal viscosity may affect CH measure. In previous studies, it has been shown to decrease CH with viscosity reduction [13]. Independent of other factors, increasing age can cause a decrease in viscosity [14].

The high rates IOP that we measured in DM patients compared to the control group may be an indication that diabetes affects corneal biomechanical measurements. As seen in other studies we have found that an inverse relationship between IOP and CH [15, 16].

Some studies show that high IOP and glaucoma may develop in diabetic patients. Bonovas *et al.* suggest that diabetic patients are at significantly increased risk of developing primary open-angle glaucoma [17].

In a study with using specular microscopy, chronic metabolic stress occurs as a result of hyperglycemia in diabetics and it can cause morphological changes in the corneal endothelium [18].

There are many publications which investigated the causes of the structural changes in the cornea in patients with diabetes. McNamara *et al.* suggest that hyperglycemia can affect water retention in the cornea and this situation can cause structural changes in the cornea [19].

In a research, mean CCT and Goldmann applanation tonometry IOP, IOPcc, and IOPg were significantly higher in diabetic patients than in healthy control subjects [20]. Similar results are observed in many studies [21, 22].

Corneal biomechanical differences seen in diabetic patients may be associated with statistically significantly higher IOP measurements. Actually, we do not know “how and why” diabetes affects corneal biomechanical measurements. Further studies are needed to answer these questions. We may only speculate or estimate for now as; it may be related to glucose level or something else and these changing may possibly affect the elasticity or viscosity of the cornea.

## CONCLUSION

The mean CH and CRF values were found less in the diabetic group. A statistically significant difference was found between the diabetic and nondiabetic groups in terms of IOP parameters. Mean IOPcc and IOPg values were found high in the diabetic group. We believe that this will provide a base that will shed light on other work needs to be done.

## Figures and Tables

**Table 1:** Demographics of Groups.

	Group 1 (Diabetics)	Group 2 (Non-diabetics)	p Values
Gender (n)FemaleMale	25 (50.0%)25 (50.0%)	26 (52.0%)24 (48.0%)	1.000
Age	63.3±9.0	61.7±11.6	0.459

**Table 2:** Mean IOPcc, IOPg, CH and CRF values of the groups.

	IOPccmean±SD(range)	IOPgmean±SD(range)	CHmean±SD(range)	CRFmean±SD(range)
Group 1 (Diabetics)	17.8±3.6(12.1 – 29.0)	16.9±3.5(10.9 – 25.9)	9.9±1.5(6.1 – 13.3)	10.4±1.6(7.5 – 14.0)
Group 2 (Non-diabetics)	16.0±3.1(10.9 – 23.8)	15.4±2.9(9.0 – 24.7)	10.5±1.7(6.5 – 15.7)	10.5±1.7(6.6 – 15.4)
p values	**0.006**	**0.032**	0.080	0.730

SD = Standard Deviation
